# Ötz-T: 3D-printed open-source turbidity sensor with Arduino shield for suspended sediment monitoring

**DOI:** 10.1016/j.ohx.2023.e00395

**Published:** 2023-01-18

**Authors:** Jessica Droujko, Felix Kunz Jr, Peter Molnar

**Affiliations:** a Institute of Environmental Engineering, ETH Zurich, 8093 Zurich, Switzerland; b Institute of Information Technology and Electrical Engineering, ETH Zurich, 8092 Zurich, Switzerland

**Keywords:** Turbidity, Data logging, Environmental sensing, Printed circuit board, Real-time clock, Rivers

## Abstract

Fine sediment production in catchments and transport through rivers to floodplains and coastal areas is extremely important for riverine, coastal and marine ecosystems, nutrient transport, global biogeochemical cycles, water quality and pollution. Due to the high cost of suspended sediment monitoring technology, it is extremely difficult to obtain a complete understanding of the physical connections between climate, hydrology, fluvial processes, and sediment fluxes, which requires measurements at many locations. For this reason, we have built an open-source turbidity sensor that brings accessibility to global river research. Compared to commercial turbidity sensors (>6000€), our low-cost version (∼200€) allows for multiple deployment and therefore a high spatial coverage of sediment fluxes. It is an optical scatter sensor with an 850 nm LED and two IR detectors, and features a temperature and pressure sensor. Our sensor is 3D-printed on a hobby printer and is programmed with Arduino IDE, making it accessible to those without high-tech workshop access and limited programming skills. It features a printed circuit board that stacks on top of an ultra low-power Arduino MKR WAN 1310, for durability and easy assembly. The sensor was tested during a flood in September 2022 on the Ötztal Ache in Tirol, Austria.

## Graphical abstract

**Figure:**
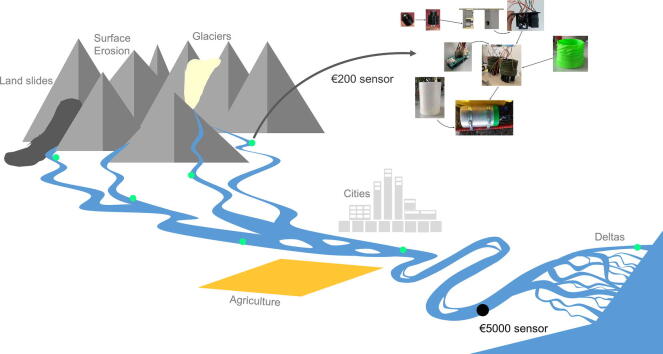

**Specifications table**

**Table:** 

**Hardware name**	Ötz-T
**Subject area**	Environmental, planetary and agricultural sciences
**Hardware type**	Field measurements and sensors
**Closest commercial analog**	In-situ turbidity sensor. https://insitu.com/en/aqua-troll-600-multiparameter-sonde
**Open source license**	GNU Affero General Public License v3.0
**Cost of hardware**	217 CHF
**Source file repository**	https://doi.org/10.5281/zenodo.7185235

## 1 Hardware in context

The transfer of sediment from land to oceans plays an important role in the global denudational cycle [19], [46], the global geochemical cycle [31], [28], the function of coastal ecosystems [36], [3], and the evolution of deltas and other coastal landforms [34], [30], [37]. Proper monitoring of this sediment flux should capture the temporal and spatial variability in concentrations at suitable timescales. This variability in suspended sediment concentrations (SSC) can then be used to quantify human effects on sediment production [45], [21], the natural erosion gradients over entire mountain ranges [20], and the uncertainty in erosion rates related to short-term sampling [10]. It can also give us a better understanding of possible short-term hydrological processes in a catchment leading to sediment production, e.g. hillslope erosion by rainfall events, glacier ice melt erosion, even hydropower storage in dams, as well as longer-term variability caused by ongoing climate change [33], [21], [8], [9]. Measurements of SSC are also important for understanding how the impacts of hydroclimatic forcing on activating sediment sources may propagate through the river system in observations and also in physically-based hydrology-sediment models [5], [6], [9], [25], [41]. However, understanding such processes requires a temporal and spatial perspective on sediment pathways of production and storage within the catchment which cannot easily be achieved by current methods and sensing technology to measure SSCs.

The primary method to quantify SSC is by gravimetric analysis of bottle samples taken at river cross-sections in regular or irregular intervals. This method is reliable but has many disadvantages such as being discontinuous, inefficient and costly. Alternatively, the spatial distribution of SSC can be obtained using satellite imagery which is based on the reflectance of water surface as it is affected by suspended sediment. Dissolved and suspended sediment concentrations together with gross biological activity affect the intrinsic colour of natural waters [35], [42], which makes optical satellite remote sensing of oceans, coastal areas, large lakes/rivers possible. When calibrated with ground measurements, such satellite data can be very useful for SSC estimates [11], [29], [14] and can give a range of additional water quality parameters [43] at large scales but not with high temporal resolutions (they are limited by the repeatability given by satellite overpasses) and with poor point accuracy. Terrestrial photography is another possibility to obtain SSCs from the optical sensing of river turbidity [18], [16], for example by mobile phone cameras [27]. However, all satellite, ground-based and UAV optical sensing methods are limited by cost, poor temporal resolution, insufficient spatial footprint, and are strongly affected by many other constraints, which make them currently not very suitable for regular long-term monitoring of SSC in rivers.

The current state-of-the-art method to obtain continuous SSC data at high temporal resolutions is by dedicated in situ turbidity sensors, where via calibration a strong relation between turbidity and SSC can commonly be ensured. The main deficiency, however, of these commercial sensors is that they are expensive (e.g. state-of-the-art turbidity sensor by Campbell is about 6000€, In-Situ is 7000€), making widespread deployment at many sites along a river system to quantify spatial variability next to impossible.

The appealing alternative are low-cost turbidity sensors, and several such sensors have been documented in peer-reviewed literature, such as the appliance-based sensors of Gillett [17] and Trevathan [40], the backscatter systems of Jiang [22] and Eidam [13], the dual-beam detectors of Lambrou [26] and Wang [44], the handheld system of Kelley [23], and the flow-through systems of Kitchener [24] and again Gillett [17]. However, none of these systems are sufficiently accurate in the entire 0–4000 Nephelometric Turbidity Unit (NTU) range and also sufficiently robust for deployment in rivers in the field.

The open-source, low-cost, in situ turbidity sensor for river network monitoring [12] which we developed in our group meets the criteria of sufficient range and accuracy, and suitability for river deployment. This sensor was calibrated for the full 0–4000 NTU range, in addition to 0–16 g/L range, and errors were quantified in laboratory tests. Its performance was also compared to other commercially available turbidity sensors. Although the first version was built for in situ river network monitoring, there were several improvements that the sensor needed to undergo before long-term, underwater deployment could be possible. In this work, we present these improvements which lead to the Ötz-T (or Ötz-Turbidity, inspired by the natural mummy, Ötzi, found in the Ötztal alps in 1991), and we test this latest version during a flood event on the Ötztal Ache in September 2022.

## 2 Hardware description

The Ötz-T sensor is a 19 cm long (9 cm diameter) 3D printed standalone device used to measure the turbidity in rivers over extended periods of time and costs ∼25 times less than comparable commercial turbidity sensors. This sensor measures turbidity using an IR LED (850 nm) and two IR detectors, and it also features a temperature and pressure sensor to obtain river temperature and stage.

**Fig. 1:**
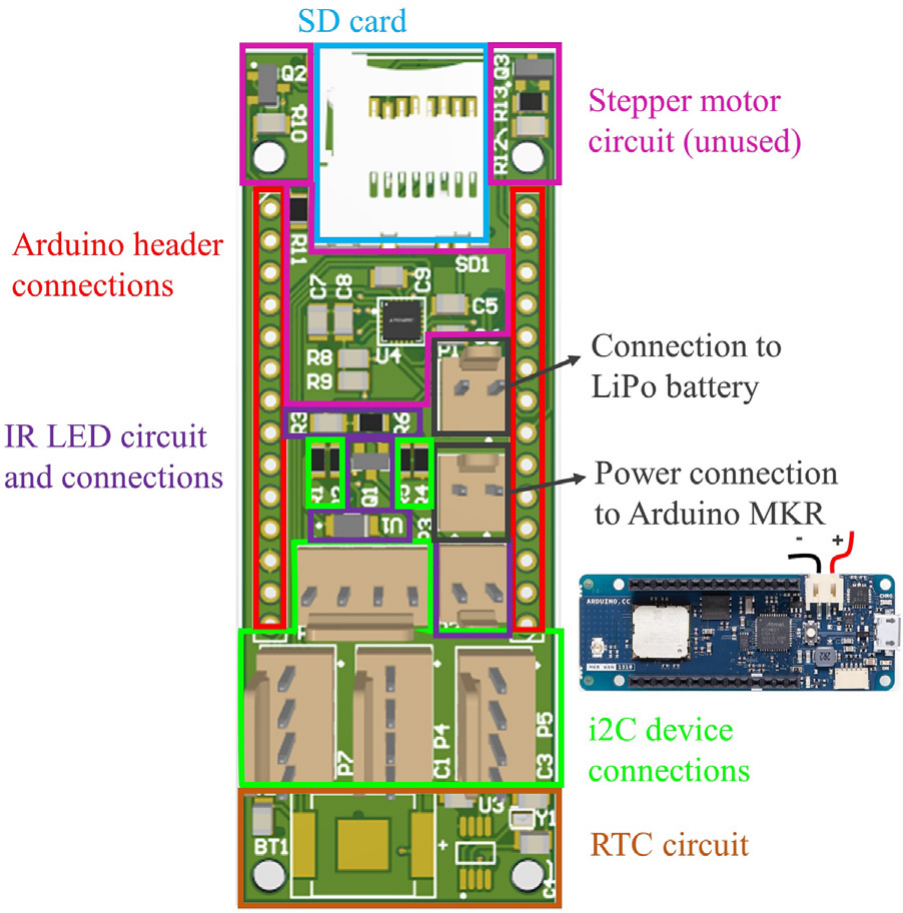
Map of the custom PCB shield. Coloured boxes have respective descriptions. This PCB should be stacked on top of the Arduino MKR WAN 1310 via the headers (red). The PCB also features a micro SD card slot (blue), battery connections to a LiPo battery and to the MKR (black), i2C connections for the pressure and IR sensors (green), an RTC circuit (orange), circuit and connection for the IR LED (purple), and an unused circuit for a potential stepper motor (pink). (For interpretation of the references to colour in this figure legend, the reader is referred to the web version of this article.)

The sensor features a custom printed circuit board (PCB) shown in Fig. 1. The PCB is robust and was built to sit as a shield on top of the ultra low-power Arduino MKR WAN 1310. The Arduino MKR WAN 1310 was chosen as the microcontroller because it has several powerful features for our application. It houses a SAMD21 chip which is optimized for low-power functionality and the MKR WAN 1310 also has LoRa built in, which we would like to utilize in future builds. Using an Arduino also enables us to program our device using the Arduino IDE, which we believe makes the project more accessible to those with less programming experience.

This shield has all the I2C connectors highlighted in green in Fig. 1. Since our IR detector has a fixed I2C address, we had to use the second I2C bus on the SAMD21. The second bus consists of the pins PA22 and PA23. It is important to note that these pins don’t have 10 k pull-up resistors built into the Arduino so we had to add them to our shield. To control the IR LED, we used a MOSFET together with an AL5809 (Fig. 1 purple highlight) which is a simple LED Driver. There is a small RTC circuit on the shield as well (Fig. 1 orange highlight). Even though the Arduino has an RTC built in, we decided to add an external one with a backup battery to keep track of time even if the Arduino is not powered. The commonly used DS3231 is very expensive and was not available to order at the time we designed the circuit. For this reason, we chose the PCF8523T which is much less expensive than the DS3231 but a bit less accurate; we observed a drift of 3 min over a month of running the sensor. A simple micro SD Card (Fig. 1 blue highlight) was used to store the data. We have also included a stepper motor circuit and controller chip on the shield (Fig. 1 pink highlight), so we can control a stepper motor which we would use as a wiper to clean the optics on the sensor from biofouling and sediment deposits. However, due to the unavailability of the selected TMC2300 chip, we couldn’t validate this circuit. The TMC2300 was chosen since it supports voltages as low as 2 V, which is ideal for our battery-powered application.

There are two ways to power the shield: either with rechargeable LiPo batteries or with non-rechargeable alkaline batteries. We chose to use a rechargeable LiPo cell, where the jumper (J1) on the MKR WAN 1310 should always be shorted in this case.

The PCB is designed in a way that it directly mounts to the Arduino. Both our PCB and the Arduino are open-source and our PCB will remain open-source and can always be found in the Zenodo repository. We also have separate PCBs for the LED, the IR sensors, and the pressure/temperature sensor. The main purpose of these PCBs is to have a simple way to mount the sensors to the casing and connect the wires.

This shield is perfect for our application, but it can also be used for a wide variety of other applications. Our highlights include:

• Low-power Arduino MKR WAN 1310
• Programmed in Arduino IDE
• Entirely 3D printed
• Turbidity, pressure, and temperature sensor
• 2 separate i2C ports on three headers
• 2 IR detectors for direct light to SSC calibration
• SD card reader
• RTC with a backup battery
• Cost effective

## 3 Design files summary

CAD files.

**Table:** 

**Design filename**	**File type**	**Open source license**	**Location of the file**
Thread	STEP	GNU General Public License v3.0	10.5281/zenodo.7185235
Cap	STEP	GNU General Public License v3.0	10.5281/zenodo.7185235
Teeth	STEP	GNU General Public License v3.0	10.5281/zenodo.7185235
LED_Zapfen	STEP	GNU General Public License v3.0	10.5281/zenodo.7185235
Detector_Zapfen	STEP	GNU General Public License v3.0	10.5281/zenodo.7185235

**Thread.step:** CAD file for the thread to be opened in any CAD software.

**Cap.step:** CAD file for the housing lid to be opened in any CAD software.

**Teeth.step:** CAD file for the teeth that hold the optical components, to be opened in any CAD software.

**LED**_**Zapfen.step:** CAD file for the LED and lens holder, to be opened in any CAD software.

**Detector**_**Zapfen.step:** CAD file for the detector and lens holder, to be opened in any CAD software.

3D printing files

**Table:** 

**Design filename**	**File type**	**Open source license**	**Location of the file**
Thread	STL & 3MF	GNU General Public License v3.0	10.5281/zenodo.7185235
Cap	STL & 3MF	GNU General Public License v3.0	10.5281/zenodo.7185235
Teeth	STL & 3MF	GNU General Public License v3.0	10.5281/zenodo.7185235
LED_Zapfen	STL & 3MF	GNU General Public License v3.0	10.5281/zenodo.7185235
Detector_Zapfen	STL & 3MF	GNU General Public License v3.0	10.5281/zenodo.7185235

**Thread.stl:** STL file for 3D printing the thread

**Cap.stl:** STL file for 3D printing the housing lid

**Teeth.stl:** STL file for 3D printing the teeth that hold the optical components (LED zapfen and Detector zapfen)

**LED**_**Zapfen.stl:** STL file for 3D printing the LED and lens holder (“zapfen”)

**Detector**_**Zapfen.stl:** STL file for 3D printing the detector and lens holder

**Thread.3mf:** PrusaSlicer file for the thread

**Cap.3mf:** PrusaSlicer file for the housing lid

**Teeth.3mf:** PrusaSlicer file for the teeth that hold the optical components (LED zapfen and Detector zapfen)

**LED**_**Zapfen.3mf:** PrusaSlicer file for the LED lens holder

**Detector**_**Zapfen.3mf:** PrusaSlicer file for the detector and lens holder

PCB files

**Table:** 

**Design filename**	**File type**	**Open source license**	**Location of the file**
MainPCB	.SchDoc &.PcbDoc & Gerber &.kicad_pcb &.kicad_sch &.pdf	GNU General Public License v3.0	10.5281/zenodo.7185235
PressureSensor	.SchDoc &.PcbDoc & Gerber &.kicad_pcb &.kicad_sch &.pdf	GNU General Public License v3.0	10.5281/zenodo.7185235
LightSensor	.SchDoc &.PcbDoc & Gerber &.kicad_pcb &.kicad_sch &.pdf	GNU General Public License v3.0	10.5281/zenodo.7185235
IRDiode	.SchDoc &.PcbDoc & Gerber &.kicad_pcb &.kicad_sch &.pdf	GNU General Public License v3.0	10.5281/zenodo.7185235
SolderPasteStencil	svg	GNU General Public License v3.0	10.5281/zenodo.7185235

**MainPCB.SchDoc:** The Schematic File of the Main PCB

**MainPCB.PcbDoc:** The PCB File of the Main PCB

**MainPCB.Gerber:** The Gerber file used to order the Main PCB

**MainPCB.kicad**_**pcb:**.PcbDoc imported to KiCad so it can be edited without Altium

**MainPCB.kicad**_**sch:**.SchDoc imported to KiCad so it can be edited without Altium

**MainPCB.pdf:** Schematic as PDF

**PressureSensor.SchDoc:** The Schematic File of the Pressure Sensor PCB

**PressureSensor.PcbDoc:** The PCB File of the Pressure Sensor PCB

**PressureSensor.Gerber:** The Gerber file used to order the Pressure Sensor PCB

**PressureSensor.kicad**_**pcb:**.PcbDoc imported to KiCad so it can be edited without Altium

**PressureSensor.kicad**_**sch:**.SchDoc imported to KiCad so it can be edited without Altium

**PressureSensor.pdf:** Schematic as PDF

**LightSensor.SchDoc:** The Schematic File of the Light Sensor PCB

**LightSensor.PcbDoc:** The PCB File of the Light Sensor PCB

**LightSensor.Gerber:** The Gerber file used to order the LightSensor PCB

**LightSensor.kicad**_**pcb:**.PcbDoc imported to KiCad so it can be edited without Altium

**LightSensor.kicad**_**sch:**.SchDoc imported to KiCad so it can be edited without Altium

**LightSensor.pdf:** Schematic as PDF

**IRDiode.SchDoc:** The Schematic File of the IRDiode PCB

**IRDiode.PcbDoc:** The PCB File of the IRDiode PCB

**IRDiode.Gerber:** The Gerber file used to order the IRDiode PCB

**IRDiode.kicad**_**pcb:**.PcbDoc imported to KiCad so it can be edited without Altium

**IRDiode.kicad**_**sch:**.SchDoc imported to KiCad so it can be edited without Altium

**IRDiode.pdf:** Schematic as PDF

**SolderPasteStencil.svg:** File to lasercut the solderpaste stencil

Software files

**Table:** 

**Design filename**	**File type**	**Open source license**	**Location of the file**
main	INO	GNU General Public License v3.0	10.5281/zenodo.7185235
set_time	INO	GNU General Public License v3.0	10.5281/zenodo.7185235

**main.ino:** firmware file to upload onto the Arduino MKR WAN 1310 and run the data logging program

**set**_**time.ino:** firmware file to upload onto the Arduino MKR WAN 1310 and set the RTC

## 4 Bill of materials summary

Sensor housing components

**Table:** 

**Designator**	**Component**	**Number**	**Cost per unit - CHF**	**Total cost - CHF**	**Source of materials**	**Material type**
Teeth	XPETG Matt 3D printing filament	56 g	0.029 chf/g	1.62	Extrudr	Polymer
Thread	XPETG Matt 3D printing filament	90 g	0.029 chf/g	2.61	Extrudr	Polymer
Cap	XPETG Matt 3D printing filament	146 g	0.029 chf/g	4.23	Extrudr	Polymer
LED Zapfen	XPETG Matt 3D printing filament	2 g	0.029 chf/g	0.06	Extrudr	Polymer
Detector Zapfen	XPETG Matt 3D printing filament	4 g	0.029 chf/g	0.12	Extrudr	Polymer
O-ring	0101–001633, NBR 70 shore, ID: 80 mm, thickness: 2.5 mm	2	1.67	3.34	Kubo Tech AG	Polymer
Epoxy	3 M Scotch-Weld DP100 Clear	1	28.62	28.62	Digi-Key	Polymer
Laqueur	UV-resistant gloss varnish	10%	9.95	0.995	Jumbo	Non-specific
Vacuum grease	Silikonfreies Laborfett glisseal HV, 60 g	10%	52.50	0.525	Borer	Non-specific

Optical components

**Table:** 

**Designator**	**Component**	**Number**	**Cost per unit - CHF**	**Total cost - CHF**	**Source of materials**	**Material type**
LED1	TSHG6200	1	1.10	1.10	Mouser	Semiconductor
U1-LS/ IR detector	TSL25911FN	2	1.74	3.48	Mouser	Semiconductor
Lenses	6 mm plano convex lens	3	2.96	8.88	Nanyang City Jingliang Optical Technology Co., LTD	Inorganic

Power components

**Table:** 

**Designator**	**Component**	**Number**	**Cost per unit - CHF**	**Total cost - CHF**	**Source of materials**	**Material type**
LiPo battery	Li-ion Rechargeable pack 3.7 V 10.4Ah	1	41.45	41.45	RS Components	Non-Specific
Coin cell battery	1.55 V coin 6.8MM	1	1.14	1.14	Digi-Key	Non-Specific

PCBs

**Table:** 

**Designator**	**Component**	**Number**	**Cost per unit - CHF**	**Total cost - CHF**	**Source of materials**	**Material type**
Pressure PCB	PCB for the MS580305 BA01	1	0.3	0.3	JLC PCB	Non-Specific
IR detector PCB	PCB for the TSL25911FN	2	0.3	0.6	JLC PCB	Non-Specific
LED PCB	PCB for the LED	1	0.33	0.33	JLC PCB	Non-Specific
Main PCB	PCB for the main board	1	0.52	0.52	JLC PCB	Non-Specific

Electrical components

**Table:** 

**Designator**	**Component**	**Number**	**Cost per unit - CHF**	**Total cost - CHF**	**Source of materials**	**Material type**
BT1	LR621	1	0.32	0.32	Mouser	Metal
C1, C2, C1-PS	100nF Capacitor	3	0.03	0.09	Mouser	Ceramic
C3, C4	10pF Capacitor	2	0.16	0.32	Mouser	Ceramic
C1-LS	1uF Capacitor	2	0.17	0.34	Mouser	Ceramic
DS1	HSMH-C190	1	0.49	0.49	Mouser	Semiconductor
P1, P2, P3, P1-LED	61900211121	4	0.44	1.76	Mouser	Non-specific
P4, P5, P6	61900411121	4	0.73	2.92	Mouser	Non-specific
Q1	2N7002LT1G	2	0.20	0.39	Mouser	Semiconductor
R1, R2, R4, R5	10 k Resistor	4	0.03	0.12	Mouser	Non-specific
R7, R3	330R Resistor	1	0.29	0.29	Mouser	Non-specific
SD1	473521001	1	3.60	3.60	Mouser	Metal
SW1	Button	1	0.56	0.56	Mouser	Non-specific
U1	AL5809-100P1-7	1	0.40	0.40	Mouser	Semiconductor
U2	Arduino MKR 1310	1	38.68	38.68	Arduino	Semiconductor
U3	PCF8523T	1	1.63	1.63	Mouser	Semiconductor
Y1	32.768KHZ	1	1.33	1.33	Mouser	Other
J1_LS, J1_PS	5–146130-1	3	0.61	1.83	Mouser	Non-specific
U1-PS	MS580305BA01	1	27.91	27.91	Mouser	Semiconductor
2 Pin Connector Female	710–61900211621	2	0.139	0.278	Mouser	Non-Specific
4 Pin Connector Female	710–61900411621	3	0.188	0.564	Mouser	Non-Specific
4 Pin Connector Female	90143–0104 (Molex)	3	0.337	1.011	Mouser	Non-Specific
Crimp Contacts	90119–2109 Crimp	12	0.098	1.176	Mouser	Metal
Crimp Contacts	61900113722DEC Crimp	16	0.141	2.256	Mouser	Metal
Wires	20AWG in Red, Green, Orange, Black	0.1 m per color	2.03/m	0.81	Mouser	Non-Specific

## 5 Build instructions

**Electronics**

**Fig. 2:**
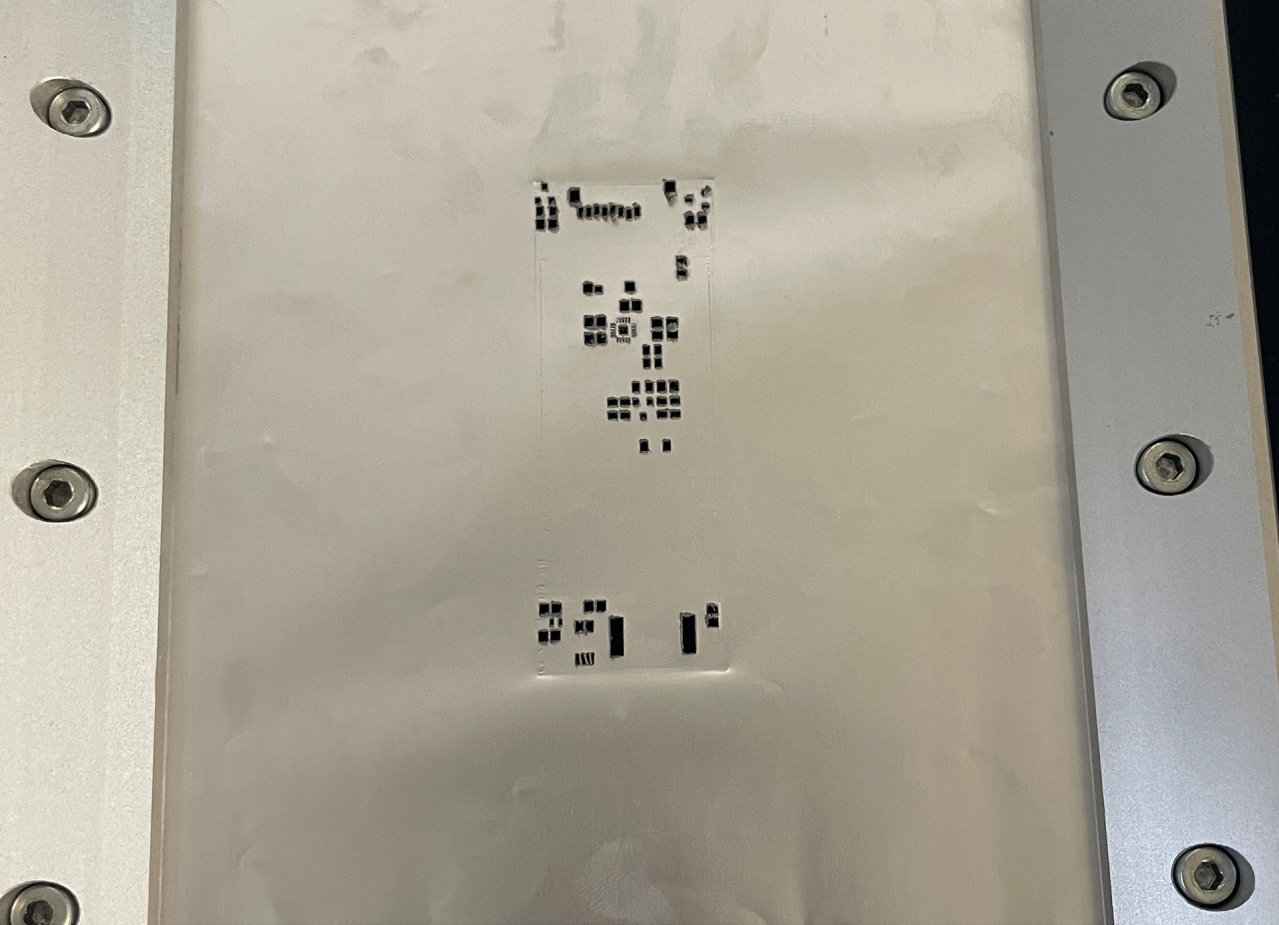
Makeshift stencil for solder-paste made with a laser cutter.

*Order the PCBs.* Order the PCBs using our Gerber Files or export your own Gerber files from Altium (the education license can be obtained for free). We used JLCPCB.com to order the boards; simply upload the Gerber files to the website. You should also select some specifications when ordering the PCB; we chose a two-layered board with FR-4 material and with a thickness of 1.6 mm. We suggest ordering the solder paste stencil (Fig. 2) but you can also create one yourself from a piece of aluminum foil and a laser cutter using our file SolderPasteStencil.svg.

**Fig. 3:**
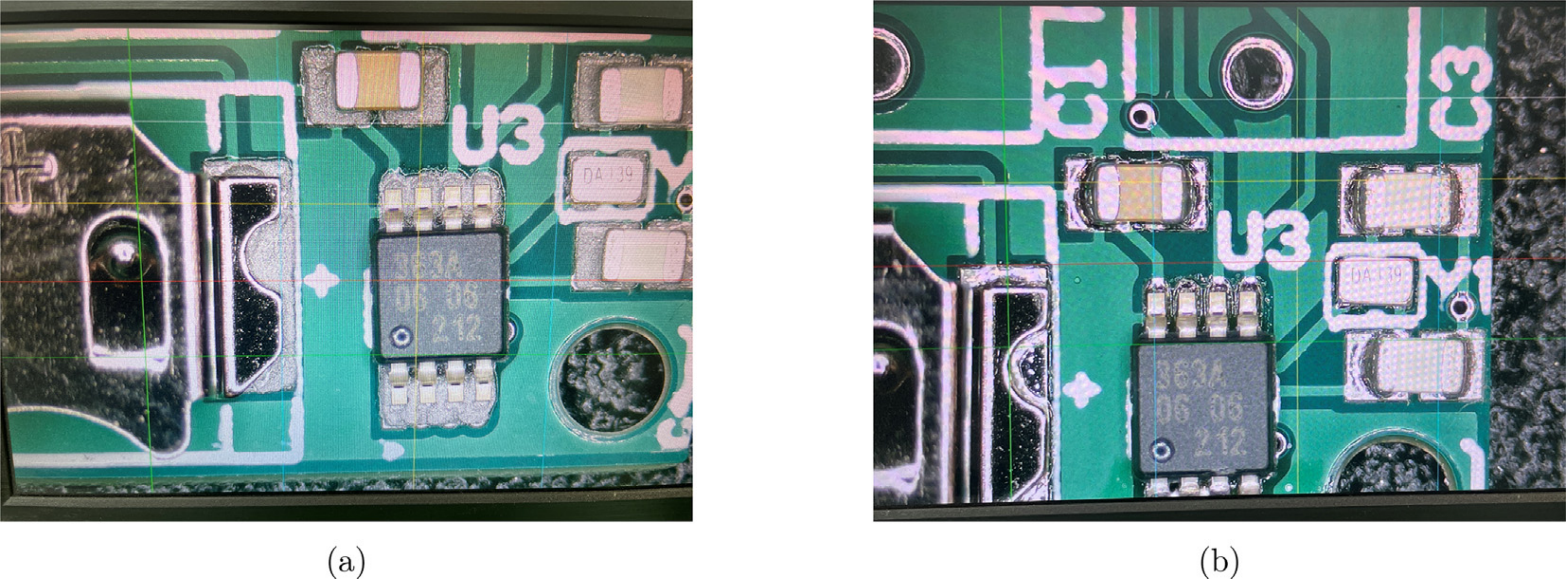
Re-flow process. a) Components placed on the PCB with solder-paste on pads, b) PCB after soldering in the re-flow oven.

*Assemble PCBs.* To assemble the PCB we used the re-flow process. After all the pads are covered with solder paste, you can place the parts on the pads according to the designators (Fig. 3a). As soon as all the components are placed and you have confirmed that they are oriented correctly, you can place the PCBs in the oven to heat them up to solder the components (Fig. 3b). It is also possible to solder the PCBs by hand. Finish the PCB assembly by adding a coat of acrylic laqueur (listed in the Bill of materials).

*Connectors.* The PCBs have many connectors to connect the different parts. All the 4 Pin Connectors, used for the I2C sensors, have the same pinout, see Table 1.

**Table 1:** Pinout and corresponding wire color of 4 Pin Connectors.

**Pin**	**Function**	**Color**
Pin 1	3.3 V	Red
Pin 2	SCL Clock	Orange
Pin 3	SDA Data	Green
Pin 4	GND	Black

The 2 Pin Connectors are used for the IR LED and the battery connections (see the 4 Pin (green) and 2 Pin (purple and black) connections in Fig. 1). Use a clamp to crimp the Molex connectors to the wires. The orientation of the connectors are marked in the Altium design files.

**Sensor housing**

*3D printing components.* Begin by 3D printing all of the.stl components listed in the Design files summary. Each component should be printed only once except the Detector_Zapfen.stl should be printed twice. For optimal printing performance, we suggest the following options when slicing the.stl files:

• “Avoid crossing perimeters” should be enabled so that there is less material to clean after printing.
• 5 perimeters, 5 bottom solid layers, and at least 0.15 mm layer height for all components.
• The lens inserts (LED Zapfen and Detector Zapfen) should have a smaller layer height of 0.07 mm.
• The Teeth have variable layer height ranging from 0.07–0.15 mm. Layer height of 0.15 mm for time optimization and a layer height of 0.07 mm around where the Zapfen would fit into the teeth.

**Fig. 4:**
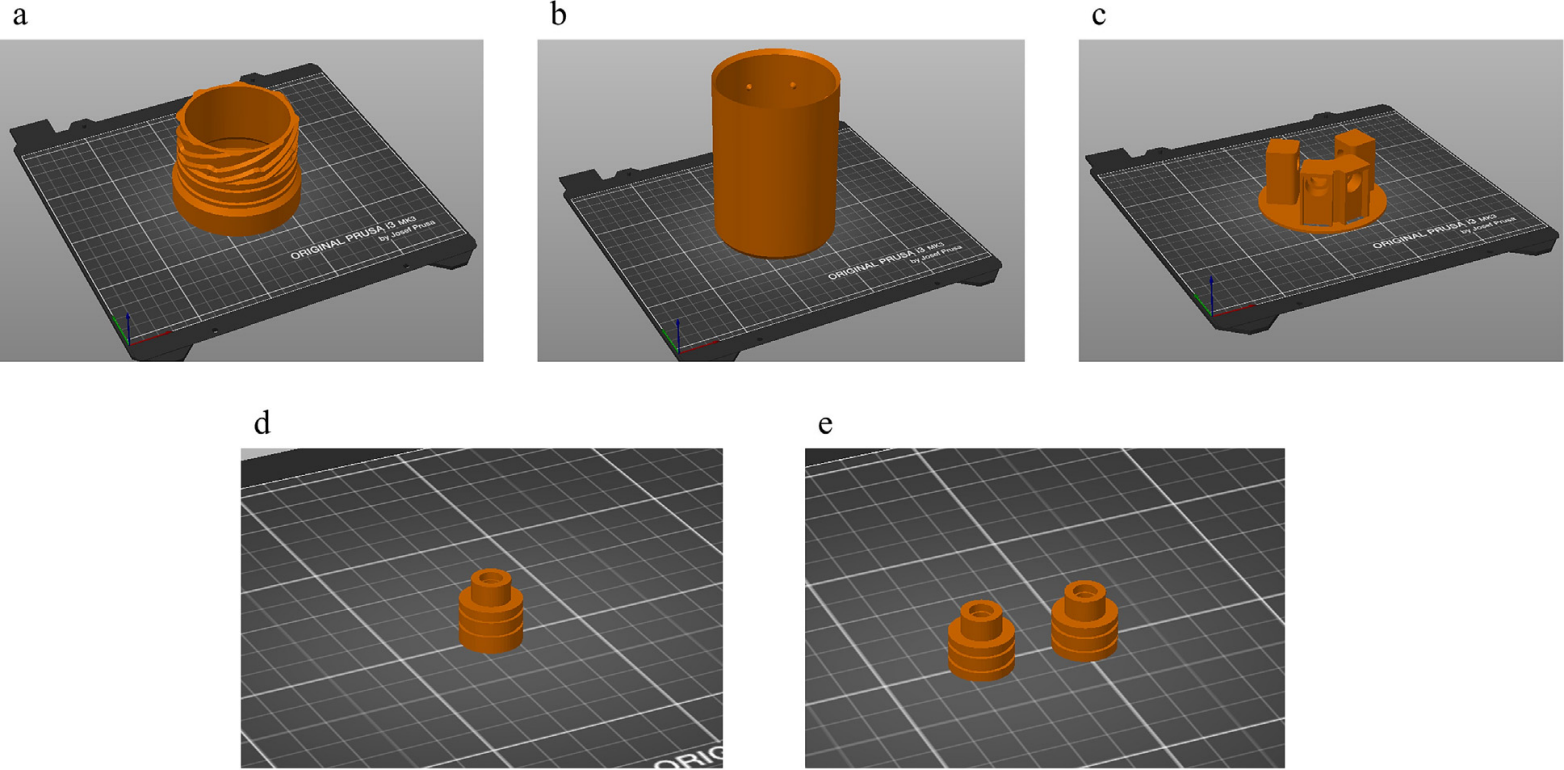
Optimal placement of components on 3D printing bed: a) thread, b) cap, c) teeth, d) LED zapfen, and e) detector zapfen.

Fig. 4 shows how to place the components on the printing bed for optimal printing. All of the components are placed on the bed to avoid overhangs and the ensure a smooth surface. The Zapfen are needed because without them it is very difficult to get a clean print in the Teeth around the lenses. Printing the Zapfen as separate components, as in Fig. 4 d and e, we get a high accuracy print that aligns the optical components to our satisfaction. We can then insert the Zapfen into the Teeth. If using Prusa I3 Mk3/Mk3S Filament Printer, we have provided the.gcode files, which can be used directly on the printer, and include all of the perimeter settings, predefined supports, and layer heights. We chose XPETG as the filament material since it does not dissolve when exposed to UV and water. Once the components have been printed, spray the Cap and Thread with acrylic laqueur (see Bill of materials). Although the components are waterproof, we have found that this helps keep water out of the walls of the 3D printed components.

**Fig. 5:**
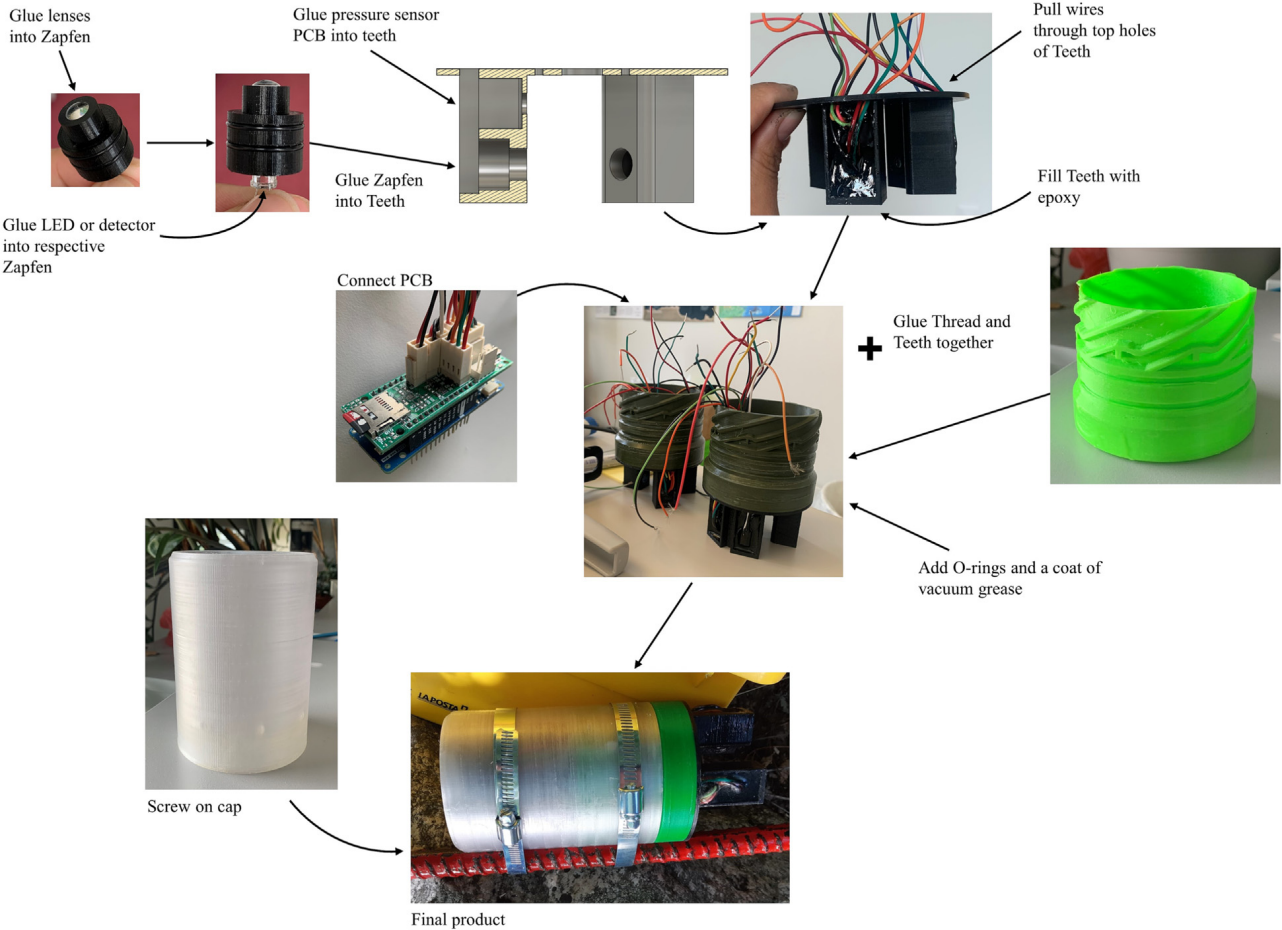
Assembly of sensor: beginning in the top left corner and following arrows until the Final Product.

*Component assembly.* Once all of the pieces are printed, assemble them as in Fig. 5. Glue the lenses into the zapfen. Once the glue is dry we suggest testing if the lenses leak since this can destroy the IR detectors. Solder wires (according to Table 1) to the back of the LED, pressure, and IR detector PCBs. Then glue the IR detector and LED PCBs into the Zapfen. Glue the three Zapfen into the Teeth (the two IR detectors should be at $45^{o}$ and $135^{o}$ relative to the LED). Afterwards, glue the pressure sensor into the Teeth. We used the epoxy listed in the Bill of materials. Feed the wires from the LED, IR detector, and pressure/temperature sensor PCBs through the holes in the Teeth. Glue the Thread and Teeth together, then fill the Teeth with epoxy. Add/crimp the Molex 2-pin and 4-pin connectors to the wires (according to Table 1). Once the epoxy is dry, add the o-rings to the Thread and coat the o-rings in vacuum grease. Finally, connect the wires to the main PCB as in the layout in Fig. 1 and the provided schematic.

## Operation instructions

Begin by uploading the first program set_time.ino to the Arduino MKR WAN 1310. Please note that you will need a USB micro B cable to connect the Arduino to your computer and you will need Arduino IDE installed [1]. Please follow the instructions in set_time.ino about changing the date in the program. After the program has flashed, every time the Arduino restarts (disconnect and reconnect to power) it will restart the RTC time to the original compile time of the set_time.ino program. To mitigate this issue, the next program, main.ino, can be immediately flashed without disconnecting power or set_time.ino can be re-flashed after commenting-out line 79 (“setPCF85263();”). Follow by flashing main.ino onto the Arduino. Please note that additional libraries may need to be downloaded and added to your library (e.g. the MS5803-05 external library [32]). The main.ino program was written to take a measurement every hour (one measurement is an average of 15 measurements) to conserve battery, but this can be changed in the code if you require higher temporal resolution to capture larger variability e.g. from flushing of sediment from a dam.

After flashing the Arduino, disconnect the USB, insert a microSD card and connect the 3.7 V LiPo battery. Once the battery is connected, the device should begin taking measurements. In order to avoid large sleep currents and accidentally using a corrupted card, we recommend using this SD card formatter[4] and testing the read-write capabilities of the cards with H2testw[38].

Once everything is running, we added some silica packets into the housing before closing the sensors for calibration, then installation.

**Calibration**

**Fig. 6:**
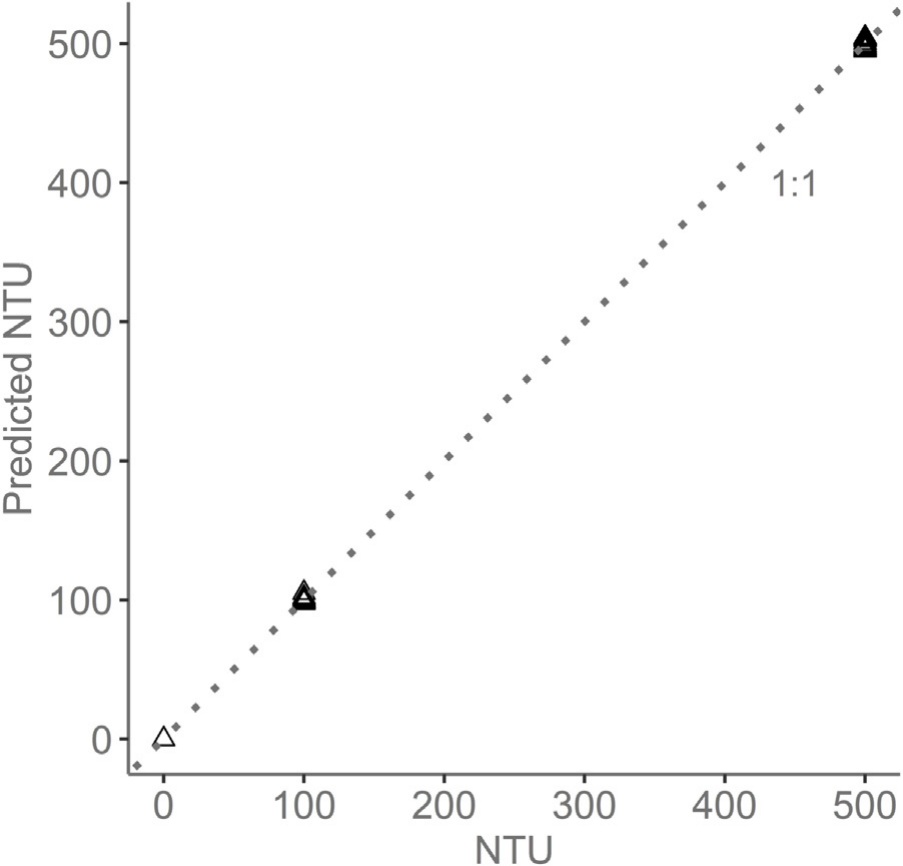
Predicted versus Observed NTU with the model. The model was calibrated using three formazin dilutions at 0, 100, and 500 NTU. The dotted line is the 1:1 line.

Before installation, we calibrated the sensor using three different dilutions of formazin: 0, 100, and 500 NTU. These three points were chosen because we wanted to limit our exposure to formazin, which is a known carcinogen, and we expected the Ötztal Ache’s turbidity to be within this range since 90% of the time this river has an SSC of <5 mg/L [7]. In addition, our last open-source sensor [12] had 12 formazin calibration points and we wanted to see if we could obtain similarly accurate data with less calibration points. The form of the model that converts the digital IR light output to NTU is:

(1) $$\mathrm{NTU}=\alpha +(\beta \times D_{\mathrm{IR}})+(\gamma \times D_{\mathrm{IR}}^{2})$$

where $D_{\mathrm{IR}}$ is the digital reading output by the IR detector’s ADC, and the calibration coefficients are listed in Table 2. The fit for the three NTU dilutions in shown in Fig. 6. The residual standard error of this model is 2.26 NTU ($\mathrm{Adj}.R^{2}=0.99$) and the P-value <2.2e-16. This model was built using only the backscattering detector, other alternatives which combine detectors at different angles were compared in [12].

**Table 2:** Model statistics.

	Coefficients	P-value
α	-1.72	0.028
β	3.49e-02	<2e-16
γ	5.46e-06	<2e-16

**Safety concerns**

LiPo batteries can be extremely dangerous if mishandled; for example, if the positive and negative terminals touch, if they are left out in the sun, and/or if a NiCd/NiMH charger is accidentally used. Please follow manufacturers safety instructions.

## 6 Validation and characterization

**Validation**

**Fig. 7:**
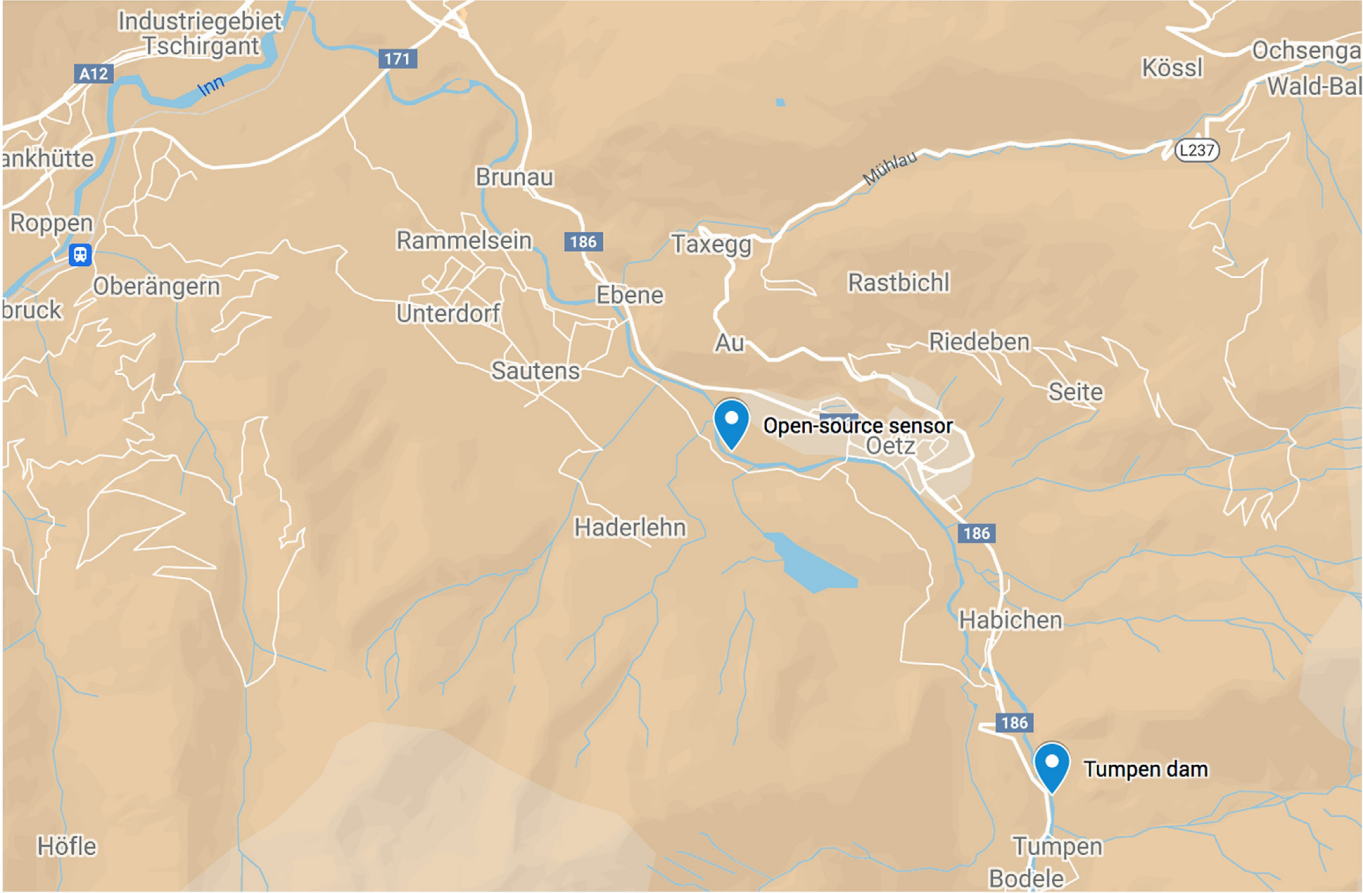
Ötztal valley, Austria. The Ötztal Ache flows North-West until it joins the Inn river. The Ötz-T sensor was located around 4.13 km downstream of the Tumpen dam.

We validated the sensor by installing it in the Ötztal Ache in Tirol over three days to measure the SSCs during the passage of a flood. The Ötz-T was installed ∼4.13 km downstream of the Tumpen dam (Fig. 7). The Ötz-T sensor took one measurement every hour from 14.09.2022 15:35 to 17.09.2022 08:24. Each measurement consists of a temperature, a pressure, and a turbidity reading (which is an average of 15 turbidity measurements, see main.ino code). The Tumpen dam data was obtained from the Hydrographische Dienst Tirol [39]; their measurements are made using a probe on the riverbank. In the following figures we are showing the measurements of the Ötz-T together with observations at the Tumpen dam upstream.

**Fig. 8:**
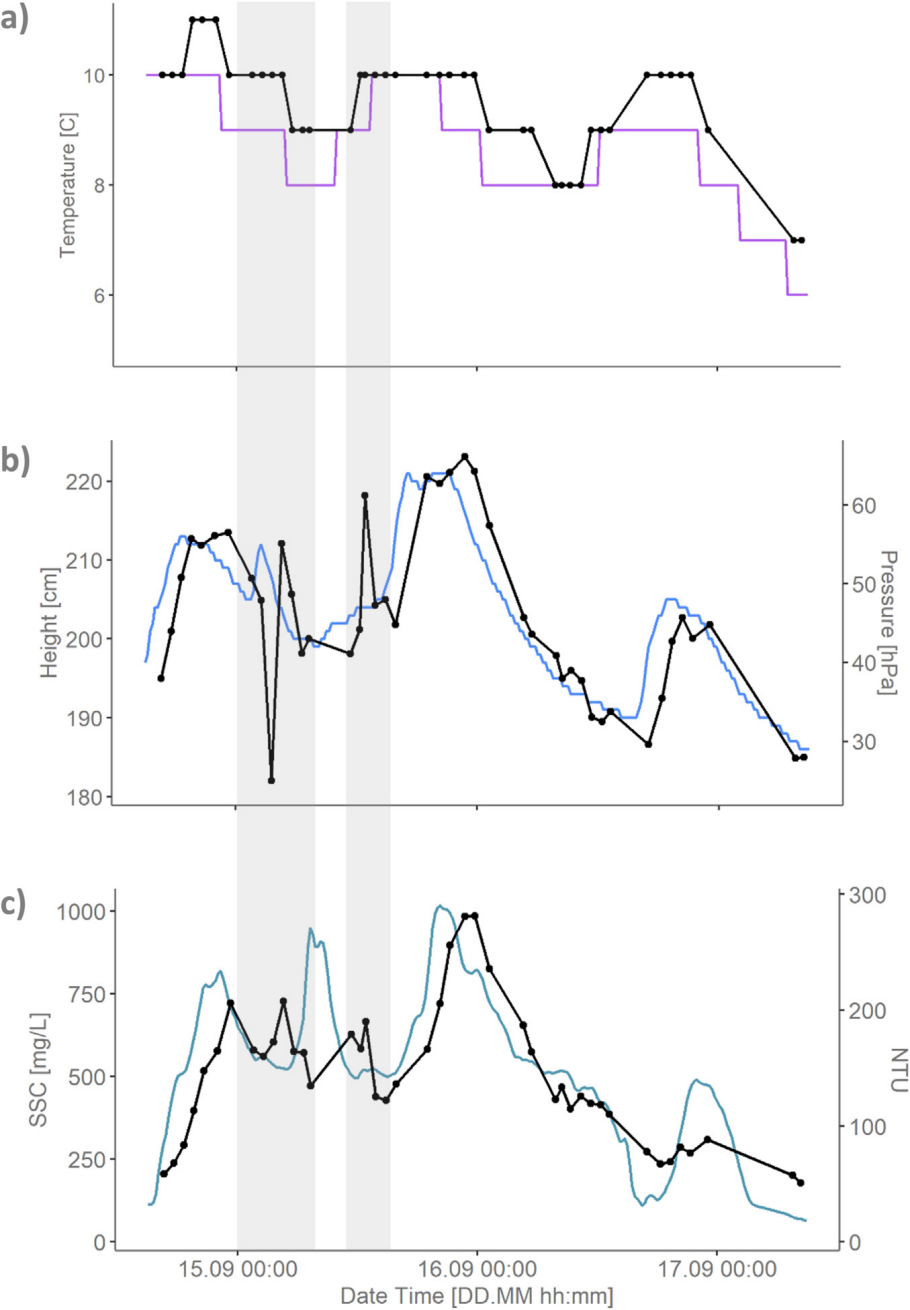
Comparison between the upstream Tumpen dam data and our downstream Ötz-T data. a) Temperature sensor comparison between Tumpen (purple) and Ötz-T (black dots and line), b) pressure sensor comparison between water level [cm] in Tumpen (blue) and Ötz-T pressure [hPa] (black dots and line) where Ötz-T pressure is the water pressure after removing the atmospheric pressure recorded at Imst, Austria, and c) comparison between SSC data [mg/L] from the Tumpen (teal) and the Ötz-T (black dots and line) turbidity data [NTU]. (For interpretation of the references to colour in this figure legend, the reader is referred to the web version of this article.)

The temperature measurements are shown in Fig. 8a. The Ötz-T data (black) seem to follow the same trend as the data from the Tumpen dam (purple). However, the Ötz-T data seems to overestimate the temperature and an offset (delay) also seems be present. Although it is difficult to conclude about the origin of the offset due to the low recording resolution, it is likely that this is a real feature related to the attenuation of the flood waves in the 4.13 km reach between the measurements. At flow velocities of 0.5–1 ${\text{m}}^{3}/\text{s}$ we may expect attenuation of 6–120 min.

The pressure measurements can be seen in Fig. 8b. The Ötz-T pressure sensor is calibrated in a vacuum and the measurements plotted in Fig. 8b (black) are the Ötz-T absolute pressure minus the atmospheric pressure recorded in Imst, Austria for the same time period. The Tumpen data (blue) is the height of the water from their measurement probe. Both measurements follow the same trends but with a shift, which again can be attributed to flow attenuation over the 4.13 km of the river reach.

The turbidity measurements are shown in Fig. 8c. The teal line is the SSC data from the Tumpen dam and the black dotted line is our Ötz-T turbidity data in units of NTU. The Tumpen SSC data is obtained via a turbidity probe on the river bank, which is then routinely calibrated to suspended sediment samples [39]. As can be seen in Fig. 8c, our turbidity data follows the trend of the SSC data (peaks and troughs) very well, again with the offset related to the delay of the sediment wave arriving to our downstream site. There are a few notable differences between the two datasets. First, around midnight on 15.09 (left grey highlight) there are two small sediment peaks in our turbidity data where there is only one large peak in the SSC data upstream. The pressure data (Fig. 8b, left grey highlight) observes a dip at the same time. Later (right grey highlight), there is again a peak in the pressure data and another NTU peak which is not as large as the SSC peak. This suggests that there can be fluctuations in the stage-SSC relationship related to short term sediment flux variations (e.g. some disturbance to the flow regime around the gauge causing a drop in stage and rise in SSC). Another explanation is that SSC and NTU usually have a relationship of the form:

(2) $$\mathrm{SSC}=a{\left(\mathrm{NTU}\right)}^{b}$$

where a and b are regression-estimated coefficients and are different for every watershed and sediment type (e.g. Costa [8] found a = 0.56 and b = 1.25 and Felix [15] found a = 0.59 and b = 1). Therefore, we cannot expect a linear relationship between the SSC and the NTU data. This same explanation can be applied to the trough and peak occurring just before 17.09. An alternative explanation could be that there were not enough formazin calibration points between 0-100NTU to properly capture the trough. Whether these peaks and troughs could not be fully captured due to the minimal calibration points of the conversion between SSC and NTU should be validated in future studies.

**Characterization**

The sensor we have presented here is an improvement from the previous open-source turbidity sensor[12]. This new version now incorporates a temperature and pressure sensor to monitor river stage and water temperature. It has an entirely 3D printed design so anyone with access to a hobby 3D printer can build the device. This sensor version now uses an Arduino MKR WAN 1310, enabling the microcontroller to reach extremely low power (104μA [2]) and with all of the i2C devices and the SD card, the sensor sleeps ∼200μA. This low current draw extends the battery life of our device from one day (the previous sensor[12]) to one month. The sensor is also now programmed in the widely adapted Arduino IDE. Finally, the customized PCB shield is more robust than the previous prototyping board used.

There are many improvements that this sensor may still undergo. First, we would like to add a motor to the device to wipe the optics from any deposited debris or biofauling. This feature is necessary if the sensor is to be used in long-term deployments. We have found that the best micro SD cards add 110μA to the device sleep current, even when the cards were idle. For this reason, in the next version we plan on using an SPI flash to store the data while also including a micro SD card slot to retrieve the data quickly in the field. Additionally, the LED driver we chose pulled 200 mA while taking measurements and significantly drained our battery. We aim to optimize this high LED current draw and improve the overall battery consumption of the device. Finally, the Ötz-T costs almost four times as much as our previous sensor version (61.37 CHF [12]). The parts of this sensor that increase the cost are the epoxy (28.62 CHF), the LiPo battery (41.45 CHF), the Arduino MKR WAN 1310 (38.68 CHF), and the MS5803-05 pressure/temperature sensor (27.91 CHF). All together these components account for 63% of the costs (or 136.66 CHF). We hope to eliminate some of these high costs by building our own microcontroller (eliminating the Arduino MKR), redesigning the teeth (to avoid the excessive use of epoxy), and switching to alkaline D-cell batteries.

Although we only used one of the two available IR detectors for our calibration and Ötztal measurements, the Ötz-T features two IR detectors. With enough gravimetric samples, the two IR detectors’ ADC readings can be directly calibrated to SSC (e.g. calibrating $D_{\mathrm{IR}}$ from eqn.1 to SSC directly). Traditional calibration needs to anyways undergo a gravimetric calibration (NTU to SSC as in eqn.2) and in this way, one would avoid the lengthy and dangerous formazin calibration step. In addition, using the $D_{\mathrm{IR}}$-SSC calibration is a better representation of the entire catchment since the IR detector response curve changes with different grain type and particle size. Therefore, calibrating to catchment gavimetric samples leads to a calibration more representative of the catchment sediment types and particle size. And using two IR detectors instead of one leads to a better model fit [12].

Our hope is that the Ötz-T sensor brings accessibility to global river research for many applications. We think that with the transparent design presented here, researchers can build and repair the instruments themselves, ultimately making the data from our world’s rivers, lakes, and oceans available to all.

## CRediT authorship contribution statement

**Jessica Droujko:** Data curation, Investigation, Methodology, Project administration, Software, Writing – original draft, Writing – review & editing. **Felix Kunz:** Methodology, Writing – original draft, Writing – review & editing. **Peter Molnar:** Supervision, Writing – review & editing.

## Declaration of Competing Interest

The authors declare that they have no known competing financial interests or personal relationships that could have appeared to influence the work reported in this paper.
